# Developing a Community of Practice to Provide Care Coordination and Address Health-Related Social Needs for Veterans Receiving Care in Community-Based Settings: Program Development and Survey Study

**DOI:** 10.2196/80654

**Published:** 2025-10-31

**Authors:** Carolyn Turvey, Natalie Suiter, Rhonda Fellows, Shawna Domeyer, Lindsey Fuhrmeister, Amanda Heeren, Kimberly McCoy, M Bryant Howren

**Affiliations:** 1 Veterans Rural Health Resource Center-Iowa City Veterans Health Administration Office of Rural Health Department of Veterans Affairs Iowa City, IA United States; 2 Carver College of Medicine Department of Psychiatry The University of Iowa Iowa City, IA United States; 3 Center for Access & Delivery Research and Evaluation VA Iowa City Health Care System Iowa City, IA United States; 4 Van Buren County Hospital Keosauqua, IA United States; 5 Crescent Community Health Center Dubuque, IA United States; 6 Carver College of Medicine Department of Internal Medicine University of Iowa Iowa City United States

**Keywords:** veterans, rural health care, health-related social needs, care coordination, US Department of Veterans Affairs, Veterans Health Administration, community care

## Abstract

**Background:**

Approximately half of US veterans receive care outside of US Department of Veterans Affairs (VA) Veterans Health Administration facilities—a proportion expected to rise due to the Promise to Address Comprehensive Toxics Act and expanded use of VA-purchased community care.

**Objective:**

This paper describes the structure and impact of the Veterans Care Coordination in Community Settings (VetCoor) program. VetCoor was implemented in 2 non-VA community health centers, and we explored setting, staffing, and treatment targets to enhance veteran care and inform broader dissemination.

**Methods:**

VetCoor embedded a coordinator within community-based, non-VA health care settings to improve veteran identification and address unmet medical needs. Coordinators also connected veterans with VA and local resources addressing health-related social needs. VetCoor included training on veteran needs and military culture. It also held a monthly community of practice call where coordinators shared best practices and met with facility representatives to learn about VA services.

**Results:**

From May 2021 to September 2023, a total of 220 veterans participated, engaging in 773 sessions. Of these 220 veterans, 73 (33.2%) received VA enrollment assistance; 54 (24.5%) were referred for medical care; and 82 (37.3%) received care coordination, including medication reconciliation assistance. They also received assistance with transportation (46/220, 20.9%) nutrition and food access (42/220, 19.1%), housing and repair (42/220, 19.1%), and utility payment support (31/220, 14.1%). Common barriers to veterans seeking care were perceptions that enrolling in the VA took resources from veterans more in need and confusion regarding discharge papers required for enrollment.

**Conclusions:**

VetCoor supported rural veterans’ health care and health-related social needs using dedicated coordinators. This model addresses resource gaps, fosters VA-community collaboration, and aligns with the VA’s expanding benefits under the Promise to Address Comprehensive Toxics Act.

## Introduction

Approximately half of all veterans receive health care outside of US Department of Veterans Affairs (VA) Veterans Health Administration facilities [1-3]. Some veterans do not enroll in the VA, whereas others enroll but seek access at both VA and community health care settings due to dual insurance coverage—referred to herein as “dual-use veterans.” Veterans enrolled in the VA may also receive community-based health care reimbursed by the VA, also known as purchased care. The VA allows veterans to seek care locally but reimbursed by the VA if they live far enough away from a VA facility or they have an extended wait for needed care. Recent programs such as the Maintaining Internal Systems and Strengthening Integrated Outside Networks Act [4] and the original Veterans Access, Choice, and Accountability Act of 2014 [5] have greatly expanded VA purchased care and, thereby, the total number of dual-use veterans.

Rural veterans often qualify for VA purchased care in the community due to the eligibility criterion based on distance from a VA facility. Nonetheless, they often find themselves in health care physician and nursing shortage areas, which is exacerbated by ongoing rural facility closures [6,7]. In light of the rural health care access crisis, the federal government provides enhanced reimbursement for federally qualified health centers (FQHCs), critical access hospitals (CAHs), or rural health clinics (RHCs). Therefore, rural veterans often combine care from the VA with local FQHCs, CAHs, or RHCs.

This paper describes the development and implementation of Veterans Care Coordination in Community Settings (VetCoor), a program designed to address cross-system coordination between the VA and rural health care facilities. VetCoor builds on work addressing the essential components of cross-system coordination [8-12], as well as previous pilot studies testing models to improve VA and rural community physician coordination [11,13-16]. The aim of the program is to develop and test novel relationships between the VA and community providers and engage veterans in the full range of their potential VA benefits.

Cross-system coordination is currently being explored within the VA as well as through models such as the relational coordination theory by Gittell et al [8] or the emphasis on relational, informational, and management continuity of care by Haggerty et al [9,17]. In this context, Miller et al [18] explored cross-system care coordination by interviewing veterans and community providers about their wishes for optimal care coordination. Veterans valued highly the dedication of VA staff yet also wanted enhanced access to local non-VA providers. Veterans reported confusion about overall responsibility for care coordination between the 2 health care systems. Community providers, when asked what would reduce such confusion, expressed their wish to have contact information for a single VA staff person highly knowledgeable in care coordination policies and procedures. They also desired contact information for the specific providers treating shared patients. Garvin et al [19], in a systematic review of dual-use care in rural settings, identified 2 policy and administrative characteristics contributing to successful interorganizational care coordination with the VA: alignment and understanding between partners and a formal contractual agreement.

Hempel et al [10] convened care coordination leadership within the VA and used a modified Delphi panel to identify research, practice, and policy recommendations for cross-system care coordination. This process yielded a recommendation for developing measures of shared care. They recommended enhanced communication technologies, determining best points of contact between health care facilities, and cross-system education about the unique cultures and patient care approaches within the 2 (or more) systems.

VetCoor aims to address many of these identified aspects of cross-system care coordination and builds on prior pilot work exploring feasible models for VA collaboration with community providers. Howren et al [14,15] and Lee et al [16] both addressed the care coordination needs of rural veterans specifically. Howren et al [14,15] improved identification of veterans seeking rural care facilities through an enhanced screening process at patient check-in, thereby improving their ability to identify shared care. The programs by both Howren et al [14,15] and Lee et al [16] developed key points of contact between the VA and community providers and provided targeted training for rural providers about veteran-specific concerns, such as military posttraumatic stress disorder or the specific needs of female veterans.

VetCoor replicates and expands upon these models, with the aim of identifying and testing new screening, staffing, and intervention parameters to increase impact while enhancing feasibility. Improving feasibility will allow for later scaling up to enroll many community providers and further enhance the reach of VA services in rural settings. Both the original pilot by Howren et al [14,15] and the VetCoor implementation detailed in this paper received funding from the Veterans Health Administration’s Office of Rural Health to enhance access to and quality of care (New Office of Rural Health Management and Analysis Database PROJ-04143).

## Methods

### VetCoor Implementation Overview

VetCoor starts with the identification of rural non-VA community providers who likely serve many veterans yet are not located close to VA medical centers. Once potential collaborative sites are identified, VetCoor leads meet with non-VA community facility leadership to discuss the benefits of VetCoor, including funding for a 0.5 full-time equivalent (FTE) care coordinator, mentorship regarding identifying patients who are veterans, and collaboration with both VA and community services to best address the needs of these veterans. If agreeable, VetCoor representatives initiate the contracting process and work with the community facility to identify potential VetCoor coordinators. Coordinator onboarding includes training about developing workflows to identify veterans in need of services, care coordination strategies for working with the VA and other community providers, veteran and military culture, and addressing health-related social needs. Coordinators are also trained on reporting requirements to convey how many veterans they are serving and what services are provided. Ongoing mentorship is provided for up to 3 years for facilities engaging in VetCoor. As part of this mentorship, coordinators participate in monthly community of practice virtual meetings where presentations by local VA staff describe VA services and coordinators share their best practices between themselves. Descriptions of each of these steps in developing VetCoor at community sites are provided in the following sections.

### Selection of Setting

Identification of potential non-VA RHCs made use of the VA geographic information systems [20,21]. Geospatial mapping allowed VetCoor leads to identify geographic regions where many veterans reside yet there are no VA facilities within 30 miles or more. Once these areas were identified, the VA could map where FQHCs, CAHs, and RHCs are located. Potential community partners were vetted to ensure that they were providers endorsed by the VA’s Care in the Community program. Endorsed providers have already been vetted by the VA for quality standards and, therefore, are eligible for reimbursement through the VA’s Care in the Community program. Once potential community partners were identified, VetCoor leads met with the VA’s local and regional strategic planners to ensure that the VA did not already have future plans to add any facility or community-based outpatient clinic in the target region.

VetCoor leads met with community facility leadership to describe the program and established the contract for the 0.5 FTE. VetCoor leads also conveyed the demands made on the coordinator during this period, including onboarding training, reporting requirements, and meeting attendance and cadence. If agreed, VetCoor initiated the contract processes with the community facility and informed the local VA of this new relationship.

This manuscript describes the implementation of VetCoor in 2 unique community-based health care settings. The first was a CAH serving a rural community with a population of 1000. This rural clinic is located 42 miles from the nearest VA community-based outpatient clinic and 86 miles from the nearest VA hospital. The second was an FQHC serving a town of 60,000 and located in the same town as a VA community-based outpatient clinic. This FQHC was located 86 miles from the nearest VA hospital. Implementation started in May 2021, and data were collected between May 2021 and September 2023.

### Identifying VetCoor Coordinators

The original pilot program by Howren et al [14,15] used nurses in a patient navigator model to assist with VA health care access and care coordination. In this replication, we explored expanding this role to include other health care delivery professionals. One of the sites participating in this project designated a nurse as the VetCoor project lead, and the other, due to local nursing staff shortages, designated a nonlicensed care coordinator. This nonlicensed care coordinator was in a career transition from law to social work but had no formal social work training at the time of the pilot. This enabled us to evaluate the feasibility and impact of engaging the designated care coordinator at the site and not insisting on nursing credentials. If successful, this would greatly improve the scalability of the VetCoor program in low-resource settings and align with other care coordination programs that use nonnursing staff such as community health workers or staff with Bachelor’s-level training [22,23].

### Onboarding Coordinators

During project orientation, coordinators met with the project leads (CT, NS, AH, and LF) to discuss implementing the veteran screen at check-in, progress in enrolling veterans, initial veteran contacts and assessment of unmet medical needs, and processes for referral for VA Care in the Community reimbursement. They were also referred to online resources educating them about VA culture, such as the VHA TRAIN Learning Collaborative program [24], or online resources provided by specific VA program offices. For example, the VA mental health services line has many online resources regarding the identification and management of posttraumatic stress and depression [25]. VetCoor coordinators were also provided with online resources describing the VA’s Care in the Community program, clarifying complex authorization processes for their health care facilities and how to determine when veterans may be eligible for VA Care in the Community.

### Community of Practice

Once a month, coordinators and VA project staff attended a community of practice meeting. These were virtual meetings as attendees were located across broad geographic settings. Each meeting addressed different subjects relevant to VetCoor, such as VA enrollment, female veterans’ health, or VA Care in the Community. A list of meetings and topics is shown in Table 1. The meetings began with brief presentations, and then coordinators were free to share best practices and address recent challenges in meeting veterans’ needs. Presenters were often from VA facilities serving the general region of the community partners to continue to build these relationships. VetCoor aimed to build strong VA facility and community health care facility ties so that when the VetCoor mentoring ended after 3 years, community providers would be fully aware of VA resources and how to collaborate with their local VA. For example, the local facility enrollment and benefits lead presented the VA enrollment policy in broad brushstrokes but then provided his contact information for any follow-up questions or if coordinators had specific enrollment questions for a veteran with whom they were working. Often, facility public relations staff or Care in the Community staff attended these meetings.

**Table 1:** Topics and timeline of community of practice presentations.

Time point	Topic	Presenter or discussant
Month 1	Introductions and presentation of the basic VetCoor^a^ structure	VetCoor lead and coordinators
Month 2	Relevant VHA^b^ TRAIN content and other online resources	VetCoor lead
Month 3	Ways of approaching veterans for VetCoor	VetCoor lead and experienced coordinators
Month 4	Understanding processes for VA^c^ enrollment	VA benefits officer from local facility
Month 5	Navigating Care in the Community referrals	Facility Care in the Community representative and experienced coordinator
Month 6	Understanding the VA formulary and formulary change requests	Facility pharmacist and experienced coordinator
Month 7	Veteran transportation benefits	Facility transportation officer
Month 8	Homeless veteran programs and resources	Facility homeless program representative
Month 9	VA women’s health	Facility women’s health program lead
Month 10	Coordinating care between the VA and the community	All current coordinators and representative patient-aligned care team nurse case manager
Month 11	Telehealth resources within the VA, including VA Video Connect, My HealtheVet, mobile apps, and patient-generated health data	Office of Rural Health telehealth expert
Month 12	Yearly wrap-up: identifying strengths and areas for improvement of the current program and community of practice model	VetCoor lead and all coordinators

^a^VetCoor: Veterans Care Coordination in Community Settings.

^b^VHA: Veterans Health Administration.

^c^VA: US Department of Veterans Affairs.

The community of practice also helped build relationships between coordinators. Discussions started during the community of practice call were often continued between coordinators after these calls without the participation of a VA representative. This allowed for candid sharing of experiences working with the VA and best practices in care coordination.

### Veteran Identification and VetCoor Participation

A multifaceted approach identified veterans in the participating community-based health care settings. First, we implemented a mandatory screening question in the community partner’s electronic health record. This health IT innovation was implemented at patient check-in for all patients’ medical visits and aided in determining which patients were veterans. The electronic mandatory screening question was as follows: “Have you served in the United States military or armed forces? This includes Air Force, Army, Coast Guard, Marines, Navy, National Guard, or Reserves.” Howren et al [14,15] developed this process to replace previous veteran screens that simply asked, “Are you a Veteran?” The latter question did not explicitly state the full range of service (eg, service in the National Guard and Reserve or service without deployment) that potentially renders one eligible for the range of VA benefits. Moreover, some veterans may not self-identify as such for personal reasons, including but not limited to if they were not in combat, do not believe that their service “counted,” or strongly believe that they do not meet income criteria for VA benefits.

VetCoor coordinators also facilitated identification of veterans by building facility and community awareness of the program. They attended routine staff meetings at their facilities, obtaining referrals from providers who identified veterans in need in the course of their regular clinical practice. Coordinators also developed relationships with their local veterans service officers and veteran service organizations such as Veterans of Foreign Wars.

Once identified, veterans were invited to meet with the VetCoor coordinator to complete an interview during which veterans were assessed for current VA enrollment status and unmet medical or health-related social needs [26,27]. When needs related to VA benefits and enrollment or other medical or health-related social needs were identified, the coordinator made and assisted with appropriate referrals. They often provided care coordination across VA and community health care providers. If health-related social needs were identified, such as food insecurity, vocational training, housing, utilities, and transportation, both VA and community resources were provided. There was no limit to the number of encounters between a VetCoor coordinator and a veteran in addressing these needs.

VetCoor coordinators uniformly recommended that all unenrolled veterans who agreed to participate in VetCoor try to enroll for VA benefits. They did so as the criteria for benefits have changed greatly with recent legislation and it is very hard to predict who is eligible and what services they may be eligible for. For example, under the Promise to Address Comprehensive Toxics Act, a veteran who is suicidal may be reimbursed for inpatient mental health treatment even if they are not enrolled in the VA at the time of admission. Coordinators addressed unmet medical needs for veterans enrolled in the VA by combining both community and VA resources and arranging for VA-reimbursed care when indicated. Among veteran participants for whom patient health care coordination with the VA was indicated, coordinators first obtained veteran release of information permissions according to their respective facilities’ health information exchange policy and practices.

If, in spite of the recommendation to enroll, veterans did not wish to apply for VA benefits, this was respected. VetCoor coordinators identified state and locally based health care and health-related social services. This included state and county-based veteran benefit offices; Medicaid if indicated; and local health, housing, and food insecurity resources. In this way, VetCoor was able to address unmet needs in veterans whom the VA was not able to assist, per veteran preference.

### VetCoor Coordinator Reporting

To facilitate the characterization of VetCoor participants and services received, a project-specific Qualtrics survey (Qualtrics International Inc) [28] was developed. Coordinators did not have experience with research data collection and, at 0.5 FTE, often held multiple roles that did not allow for extensive reporting requirements. The Qualtrics survey was developed with input from the coordinators to ensure that it was not too burdensome yet captured key components of their meetings with veterans. Using a secure link to the VA, the coordinators reported encounter-specific, nonidentifiable demographic data and a description of the services provided to the participating veteran. The survey comprised radio buttons, drop-down menus, and open-text boxes.

### Veteran Participant Characteristics

To protect veteran anonymity in reporting data to the VA, veterans were identified using unique ID numbers only, and VA program staff did not have the ability to cross-reference the ID to a veteran’s name or any VA medical records. Initial contact and return encounter dates were indicated by fiscal quarter (3-month period within a year) rather than exact encounter dates. Age ranges were reported in 10- to 20-year increments, not as the actual age or birth date. Sex, race, and ethnicity were collected.

### Program and Service Use Characteristics

Coordinators provided a narrative account of unique and return encounters with veterans via an open-text field. Narratives could include but were not limited to location of the encounter (ie, via phone or in person), participants in the encounter (ie, veteran, spouse, or physician), presenting concern (ie, need for medical or social service or VA benefit eligibility), actions taken (ie, care coordinated or referral made), and unmet needs that would require further follow-up. Narratives could be of any length.

### Analysis of Coordinator Narratives

Veteran participation data were analyzed using descriptive statistics and inductive and deductive coding of the narratives. Given that data were collected over the course of 17 months, analysis took place in phases. In the first analysis, 3 VA project team members (CT, LF, and AH) used inductive reasoning and together reviewed 30 narratives to determine activities that took place in the VetCoor encounters. These activities were added to a codebook, and consensus was reached across all 30 narratives. The analysts then divided the remaining narratives and individually coded the activities, adding codes to the codebook if new activities were identified. After individual coding was completed, the analysts met to discuss unclear narratives and reach a consensus on codes. The resulting code list was thematically analyzed, and the final codes (activities) were organized into 2 categories: health care–related needs addressed and health-related social needs addressed. For the remainder of the project, narratives were coded using deductive reasoning based on the established codebook by project team members LF and AH.

### Ethical Considerations

This program was determined to constitute quality improvement and deemed not human subject research through review by the University of Iowa Human Subjects Review Board and formal written participant consent was not required. This manuscript adheres to the Standards for Quality Improvement Reporting Excellence guidelines for qualitative reporting. Data will be destroyed at the close of the study except for what is required to be kept in accordance with the National Archives and Records Administration published in the Veterans Health Administration’s Record Control Schedule 10-1.

## Results

### Veteran and VetCoor Use Characteristics

From May 2021 to September 2023, a total of 220 veterans were identified via an embedded electronic health record screen, referral from providers within participating sites, and veteran self-referral. Veterans participating in this program engaged in a total of 773 sessions with VetCoor coordinators (Table 2). A session was considered to be any visit between the VetCoor coordinator and a veteran addressing VA benefits, health-related issues, or health-related social needs. Sessions were conducted in person or via phone or video technologies. The demographics of the participants reflect the general veteran population in rural Midwest regions, with most being male, White or non–Hispanic/Latino, and aged ≥55 years. In total, 45% (99/220) of the participants resided in rural or highly rural areas.

**Table 2:** Veterans Care Coordination in Community Settings number of unique veterans and number of sessions by age, gender, race, ethnicity, and rurality.

Characteristic		Unique veterans (n=220), n (%)	Sessions (n=773), n (%)	Ratio of sessions per unique veteran
**Age (years)**				
	17-35	16 (7.3)	47 (6.1)	2.94
	36-55	43 (19.5)	167 (21.6)	3.88
	56-65	80 (36.4)	352 (45.5)	4.40
	66-75	43 (19.5)	124 (16.0)	2.88
	≥76	38 (17.3)	83 (10.7)	2.18
**Sex**				
	Female	31 (14.1)	132 (17.1)	4.26
	Male	187 (85)	638 (82.5)	3.41
	Did not endorse male or female	2 (0.9)	3 (0.4)	2.00
**Race**				
	American Indian or Alaska Native	1 (0.5)	3 (0.4)	3.00
	Asian	1 (0.5)	2 (0.3)	2.00
	Black	7 (3.2)	23 (3.0)	3.29
	Multiracial	2 (0.9)	15 (1.9)	7.50
	White	207 (94.1)	728 (94.2)	3.52
	Preferred not to answer	2 (0.9)	2 (0.3)	1.00
**Ethnicity**				
	Hispanic or Latino	1 (0.5)	10 (1.3)	10.00
	Non–Hispanic or Latino	217 (98.6)	761 (98.4)	3.51
	Preferred not to answer	2 (0.9)	2 (0.3)	1.00
**Rurality**				
	Rural or highly rural	99 (45.0)	307 (39.7)	3.10
	Urban	118 (53.6)	462 (59.8)	3.92
	Unknown	3 (1.4)	4 (0.5)	1.33

### VA Enrollment

As shown in Table 3, half (110/220, 50%) of the participants were not enrolled in the VA when they joined the program. Of those 110 participants, 73 (66.4%) were open to discussing enrollment options. Some opted out of enrollment due to a strong belief in their ineligibility (5/110, 4.5%), whereas others declined based on personal preference (3/110, 2.7%).

**Table 3:** Health care–related and care coordination tasks addressed.

Task addressed				Unique veterans (n=220), n (%)		Sessions per task (n=773), n (%)
**VA^a^ enrollment**						
	Not enrolled in the VA		110 (50)		341 (44.1)	
	**Follow-up for those not enrolled N (% of unenrolled unique veterans and sessions)**					
		Agreed to discuss enrollment	73 (66.4)		126 (36.9)	
		Initiated enrollment	27 (24.5)		30 (8.8)	
		Declined; veteran believed they were ineligible	5 (4.5)		7 (2)	
		Declined; veteran did not wish to enroll in the VA	3 (2.7)		3 (0.1)	
**Medical referral N (% of total sample [N=220] and % of total sessions [N=773])**						
	Referred for medical care to address unmet needs		54 (24.5)		109 (14.1)	
	**Settings the veteran was referred to**					
		Referred for Care in the Community purchased by the VA	41 (18.6)		69 (8.9)	
		Referred for Care in the Community that was not reimbursed by the VA but paid for through other means	22 (10.0)		29 (3.8)	
		Referred to care at local VA facility	7 (3.2)		12 (1.6)	
**Care coordination N (% of total sample [N=220] and % of total sessions [N=773])**				82 (37.3)		158 (20.4)
	Between VA and community facility		21 (9.5)		27 (3.5)	
	Coordination of services within the community facility		59 (26.8)		94 (12.2)	
	Coordination with other non-VA community providers		19 (8.6)		33 (4.3)	
	Coordination with home health		9 (4.1)		14 (1.8)	
	Medication reconciliation		20 (9.1)		33 (4.3)	

^a^VA: US Department of Veterans Affairs.

### Medical Care Referrals

Among medical care referrals, 18.6% (41/220) were directed to the Care in the Community program. A total of 10% (22/220) were referred to non-VA community providers with costs covered by the veteran’s other insurance or out-of-pocket payments. In total, 3.2% (7/220) of the veterans were referred back to the VA for care.

### Care Coordination

Coordination efforts were primarily with providers located in the same community-based facilities as our coordinators (59/220, 26.8%). A total of 9.5% (21/220) of the participants received care coordination that facilitated a combination of community and VA care.

### Health-Related Social Needs

Many veterans indicated health-related social needs (Table 4). The most common needs included transportation (46/220, 20.9%), food insecurity (42/220, 19.1%), housing repairs (42/220, 19.1%), and utility assistance (31/220, 14.1%). In addition to facilitating enrollment in VA benefits, coordinators assisted in applications for Medicaid (20/220, 9.1%) and social security (16/220, 7.3%).

**Table 4:** Health-related social needs addressed.

Need addressed	Unique veterans (n=220), n (%)	Sessions (n=773), n (%)	Number of sessions per health-related social need, mean (SD)
Transportation	46 (20.9)	95 (12.3)	2.1 (1.6)
Food insecurity	42 (19.1)	99 (12.8)	2.4 (1.9)
Housing repair and expenses	42 (19.1)	55 (7.1)	1.3 (0.8)
Utility payment assistance	31 (14.1)	79 (10.2)	2.5 (3.9)
Applying for Medicaid	20 (9.1)	25 (3.2)	1.2 (0.4)
Housing or homelessness assistance	19 (8.6)	56 (7.2)	2.9 (3.6)
Applying for social security	16 (7.3)	46 (6.0)	2.9 (3.7)
Access to legal assistance	11 (5.0)	18 (2.3)	1.6 (1.0)
Reducing medical debt	9 (4.1)	13 (1.7)	1.4 (0.7)
Assistance with job seeking	8 (3.6)	12 (1.6)	1.5 (0.9)
Assistance with family and dependent needs	2 (0.9)	3 (0.4)	1.5 (0.7)
Education benefits	1 (0.5)	1 (0.1)	1.0 (—^a^)

^a^Not applicable.

### Recurring Issues in Care Coordination and Assisting Veterans

#### Care in the Community Reimbursement Denials

Early meetings between VetCoor VA program staff and community health care partners included representatives from the health care partners’ clinical leadership as well as administrative and billing representatives. In conducting this program, key areas of confusion arose regarding VA reimbursement for community-based care. For example, one common area of confusion was VA reimbursement for postacute care in the community. Community partners often assumed that VA authorization for an acute admission in the emergency department or an inpatient visit extended to any prescribed postacute care, such as follow-up primary care visits and diagnostic or rehabilitation services. This is not accurate as separate authorization of postdischarge care is required. This type of information was disseminated in our regular coordinator meetings to prevent costly mistakes in which often the veteran is left responsible for an unexpected medical cost.

#### Veteran Reluctance to Enroll in the VA

In light of the continually evolving legislation regarding veteran eligibility for benefits, our program strongly encouraged all interested unenrolled veterans to apply for VA benefits. One of the biggest barriers for veterans was the belief that others were more deserving of VA benefits. Coordinators explained to veterans that their enrolling in the VA did not take this opportunity away from other veterans and, in fact, it often led to greater support for the local VA. Discharge status was reviewed with veterans for accuracy, and if it was not accurate, VetCoor coordinators assisted in the process of getting the discharge status reviewed and possibly changed. Coordinators also emphasized the potential VA benefits across the life span. They reminded veterans who were currently healthy that they may at some point need the extensive wraparound services for older veterans available through the VA’s geriatrics and extended care services. These benefits are of great assistance to veterans’ loved ones as well as veterans themselves. Such arguments appealed to veterans’ value of service and putting others before themselves.

#### Administrative Barriers to Veteran Enrollment

Both the local veterans service officers and the VetCoor coordinator assisted with completing the 1010EZ form—the VA’s application for benefits—and putting together the required documentation. For example, many veterans identified in our program needed ongoing assistance related to their DD214 forms, which summarize a military service member’s duty service and related information, including discharge status. Some veterans had lost their DD214 and were not aware of how to obtain a replacement form, whereas others believed that the discharge status on the DD214 was incorrect. VetCoor coordinators assisted the veterans in addressing these issues, including applying for a discharge status review when indicated.

#### Veterans Choosing Not to Enroll in the VA

VetCoor assisted all veterans regardless of VA enrollment or eligibility status. For those who preferred non-VA care resources, coordinators referred them to programs from both public agencies and nonprofit organizations. In addition to government-sponsored programs such as Medicaid, many states have veteran relief funds such as the Veterans’ Trust fund in Iowa or the Military Family Relief Fund in Indiana. Moreover, cities, counties, and states often have programs, either publicly or privately funded, to assist with utilities, rent, housing or car repair, and access to fulfill basic needs. Coordinators turned to these resources while conveying that VA enrollment would be available at a later time if the veteran changed their mind.

## Discussion

### Principal Findings

VetCoor successfully replicates and expands upon the program by Howren et al [14,15] in addressing key components of cross-system care coordination. First, community providers needed to better assess whether a patient was a veteran. The enhanced screen embedded in the check-in process accomplished this. Community outreach, local presentations, and physician referrals also identified veterans who would then present for care coordination services at their local health care facility. Through VetCoor, coordinators were trained on VA as well as military culture. This included extensive training on the VA’s Care in the Community program and reimbursement processes. Personal relationships were forged between the community providers and key contacts at their local VA facility through ongoing care coordination combined with the community of practice meetings.

Monthly community of practice meetings educated coordinators on the VA’s housing, transportation, suicide prevention, women’s health, and Care in the Community programs. VetCoor coordinators accessed these services and also tapped into state and county resources, building up a safety net for both enrolled and unenrolled veterans. No less importantly, the community of practice allowed coordinators to share frustrations as well as best practices. For example, these meetings helped identify areas of confusion, such as postacute care authorizations or navigating the VA formulary.

A significant addition to VetCoor relative to the programs by Howren et al [14,15] and Lee et al [16] was the focus on veteran health-related social needs. The VA is currently expanding eligibility criteria and benefits addressing housing, transportation, and vocational needs. Aligning with community providers who primarily treat underserved patients enabled us to identify both enrolled and not enrolled veterans with needs related to food security, transportation, and housing. VetCoor then worked with veterans and both VA and community systems to identify resources to improve the quality of life for these veterans.

Finally, community providers engaging in VetCoor often focused on VA purchased care as a key role of VetCoor coordinators. However, this recent iteration emphasized how VetCoor’s goal is to connect veterans with a broad array of both community and VA benefits and integrate the convenience of local care with the documented high-quality care available at VA medical centers [29-32]. For example, the recommendation for all unenrolled veterans identified in our program to apply for VA benefits was based on current and future legislation that aims to broadly expand veteran eligibility for services. Under the Comprehensive Prevention, Access to Care, and Treatment Act, beginning in January 2023, veterans in an acute suicidal crisis can receive up to 30 days of inpatient care and 90 days of outpatient care regardless of VA enrollment status. Similarly, the VA’s housing benefits through the Department of Housing and Urban Development–VA Supportive Housing have significantly expanded eligibility to include veterans who served just 1 day in the military. Combining both community and VA resources is necessary in light of dwindling wraparound services in rural areas, yet such collaboration runs counter to more competitive models seen in health care systems vying to remain solvent.

### Limitations

While in development, this program faces significant limitations. Data collection remains limited due to the time demands for our coordinators practicing in busy community settings that do not necessarily have a tradition of research data collection. The data presented are based on coordinator self-report and global coding of narratives, which significantly limits the methodological rigor of this study. As this was the initial pilot phase of the study, we limited burdensome research data collection to increase the likelihood of community partner participation. Therefore, full evaluation of the program remains descriptive at this time. Currently, we are addressing this shortcoming in new sites embedded within a larger health care system where research resources are more readily available. We are planning data extraction from the medical record combined with repeated standard assessment data collected from program participants.

### Future Directions

VetCoor is expanding to 5 new sites in the Upper Midwest using both nurse and non–clinically trained care coordinators. Coordinators from previous sites who have now completed the initial 3-year contract remain engaged and available to new coordinators for training and to participate in the community of practice meetings. As we expand to new sites, we are exploring more sophisticated data collection methods that do not increase coordinator workload, such as data use agreements regarding medical record data and brief administration of standardized measures of satisfaction, health, and well-being at standard time points. We are also interviewing stakeholders directly about their experience. In this context, we are developing evaluation metrics based on the reach, effectiveness, adoption, implementation, and maintenance framework [32]. Preliminary analyses will explore whether participating veterans improve on brief measures of overall self-reported health and whether the quality of care received improves according to standard measures of quality. Veteran experience and barriers to and facilitators of broader expansion will also be collected using qualitative methods.

### Conclusions

Recent legislation (Promise to Address Comprehensive Toxics and Comprehensive Prevention, Access to Care, and Treatment Acts) extends VA benefits to Vietnam War, Gulf War, and post-9/11 veterans and those exposed to military toxins. This increases the likelihood that more veterans will turn to the VA for care—whether within VA facilities or through care purchased by the VA from community providers. The VA and community providers are facing enormous challenges meeting the needs of their communities in the context of physician and nursing shortages. VetCoor promotes enhanced collaboration and communication, with a joint focus on building a network to support veterans with extensive needs both within and outside VA purview. Future program iterations will explore integrated continuing medical education, cross-system triage, and wraparound services.

## Abbreviations

CAH: critical access hospital
FQHC: federally qualified health center
FTE: full-time equivalent
RHC: rural health clinic
VA: Veterans Health Administration
VetCoor: Veterans Care Coordination in Community Settings

## Data Availability

The datasets generated or analyzed during this study are not publicly available as their collection and use are governed by the terms of funding provided by the US Department of Veterans Affairs, which imposes restrictions on data access and distribution. Questions about specific aspects of the data could be submitted to the primary author (CT).
